# Supplementary material for: Automatic speaker diarization for natural conversation analysis in autism clinical trials
Source: Sci Rep. 2023 Jun 24;13:10270. doi: 10.1038/s41598-023-36701-4 (PMC10290724; doi:10.1038/s41598-023-36701-4)
Supplement: Supplementary file 1 — Supplementary Information. [file 41598_2023_36701_MOESM1_ESM.docx]

# Supplementary

## Inclusion and Exclusion Criteria

The inclusion criteria for participants with ASD were as follows:

- Diagnosis of ASD based on the Diagnostic and Statistical Manual of Mental Disorders (DSM-5), and the Autism Diagnostic Observation Schedule (ADOS-2).
- Children's Yale-Brown Obsessive Compulsive Scale modified for ASD (CY-BOCS-ASD) total score of at least 12.
- Clinical Global Impression-Severity (CGI-S) score of at least 4 about participant's current autism severity.
- Intelligence quotient (IQ) score of 50 or above as assessed by the Abbreviated Intelligence Quotient (ABIQ) SB5 scale.
- English proficiency compatible with the study measurements as judged by the investigator.
- Hearing, vision, and speech compatible with the study measurements as judged by the Investigator.
- All medications and treatments were expected to be stable for the duration of the study.

The diagnostic evaluations were completed at the study site by research staff and supervised by a licensed psychologist.

The exclusion criteria for all participants were as follows:

- Participation in an investigational drug or device study within 4 weeks, or five times the half-life (if it is a drug study) of the investigational molecule (whichever is longer), prior to screening and the participant is expected not to enrol in any other trial during the study.
- Co-occurring disease or condition that could interfere with, or treatment which might interfere with, the conduct of the study, or that would, in the opinion of the Investigator, pose an unacceptable risk to the participant in this study.
- Unstable or uncontrolled clinically significant psychiatric and/ or neurological disorder that may interfere with the objectives of the study.
- Participants with known "syndromic" ASD (e.g., Fragile-X syndrome, Angelman Syndrome, Prader-Willi, Rett's syndrome, tuberous sclerosis, Dup15q syndrome).
- History of alcohol misuse and/ or illicit drug use during the last 12 months prior to screening.

The inclusion criteria for the study partners were as follows:

- A staff member of the residential home can be the caregiver if this person spends sufficient time with the participant. In the opinion of the Investigator, the caregiver must be able to reliably assess the participant's mental status, activities, and behavior, and report on the participant's adherence and health. This would normally be possible when the caregiver spends a few hours each day with the participant.
- A family member living at the participant's home can be the caregiver if the participant returns home every night. When the participant returns home only over the weekend, a family member can only be the caregiver if they have intensive interaction with the participant during the week e.g., via phone calls, calls via Skype, SMS messages, etc. The quality of these interactions between caregiver and participant needs to be assessed for each participant to determine whether they are sufficient.

| **Cohort** | **Sex** | **Count** | **Age** | | **IQ** | |
| --- | --- | --- | --- | --- | --- | --- |
|  |  |  | *Mean* | *STD* | *Mean* | *STD* |
| ASD (IQ<70) | Female | 2 | 12.0 | 8.5 | 57.0 | 9.9 |
|  | Male | 20 | 16.7 | 6.9 | 59.3 | 6.6 |
| ASD (IQ>70) | Female | 8 | 17.8 | 6.9 | 80.9 | 8.5 |
|  | Male | 24 | 15.0 | 8.1 | 91.9 | 13.9 |
| NTC | Female | 9 | 17.2 | 12.8 | 100.7 | 12.5 |
|  | Male | 7 | 11.7 | 4.2 | 97.4 | 10.9 |

#### **Supplementary Table 1: Participant Demographics**

Additional demographic information showing the sex, age, and IQ of each cohort. ASD = autism spectrum disorder. NTC = neurotypical control. In addition, the ethnicity breakdown across all cohorts is as follows: 72.2% white, 7% Asian, 18% black or African American, and 2.7% multiple.

| **Threshold** | ***N* participants** | **F1 Score** | **Pearson’s r,p** |
| --- | --- | --- | --- |
| 0.50 | 33 | 0.70 | r=0.82, p=6x10^-9^ |
| 0.55 | 33 | 0.72 | r=0.82, p=7x10^-9^ |
| 0.60 | 34 | 0.72 | r=0.81, p=5x10^-9^ |
| 0.65 | 34 | 0.72 | r=0.81, p=5x10^-9^ |
| 0.70 | 34 | 0.72 | r=0.81, p=4x10^-9^ |
| 0.75 | **35** | 0.71 | r=0.81, p=3x10^-9^ |
| 0.80 | **35** | 0.70 | r=0.79, p=2x10^-8^ |
| 0.85 | **35** | 0.69 | r=0.79, p=2x10^-8^ |

####

#### **Supplementary Table 2: Cosine Distance Threshold Selection**

***Threshold:*** *The threshold (left column) for the cosine distance metric between the participant’s Speaker ID embedding and the embeddings of the speakers automatically identified by the diarization algorithm (see Figure 1). N participants: The number of participants successfully diarized (out of 35). Participants can have conversations that are undiarizable if the distance between the embedding of their speaker ID audio and the embeddings of the utterances during the conversation never go below the threshold. F1 Score: The accuracy at identifying the participant from the conversation recordings (averaged across all participants; see Figure 2). Pearson’s r,p:* *The correlation between the true and predicted mean UD (see Figure 3).*

*
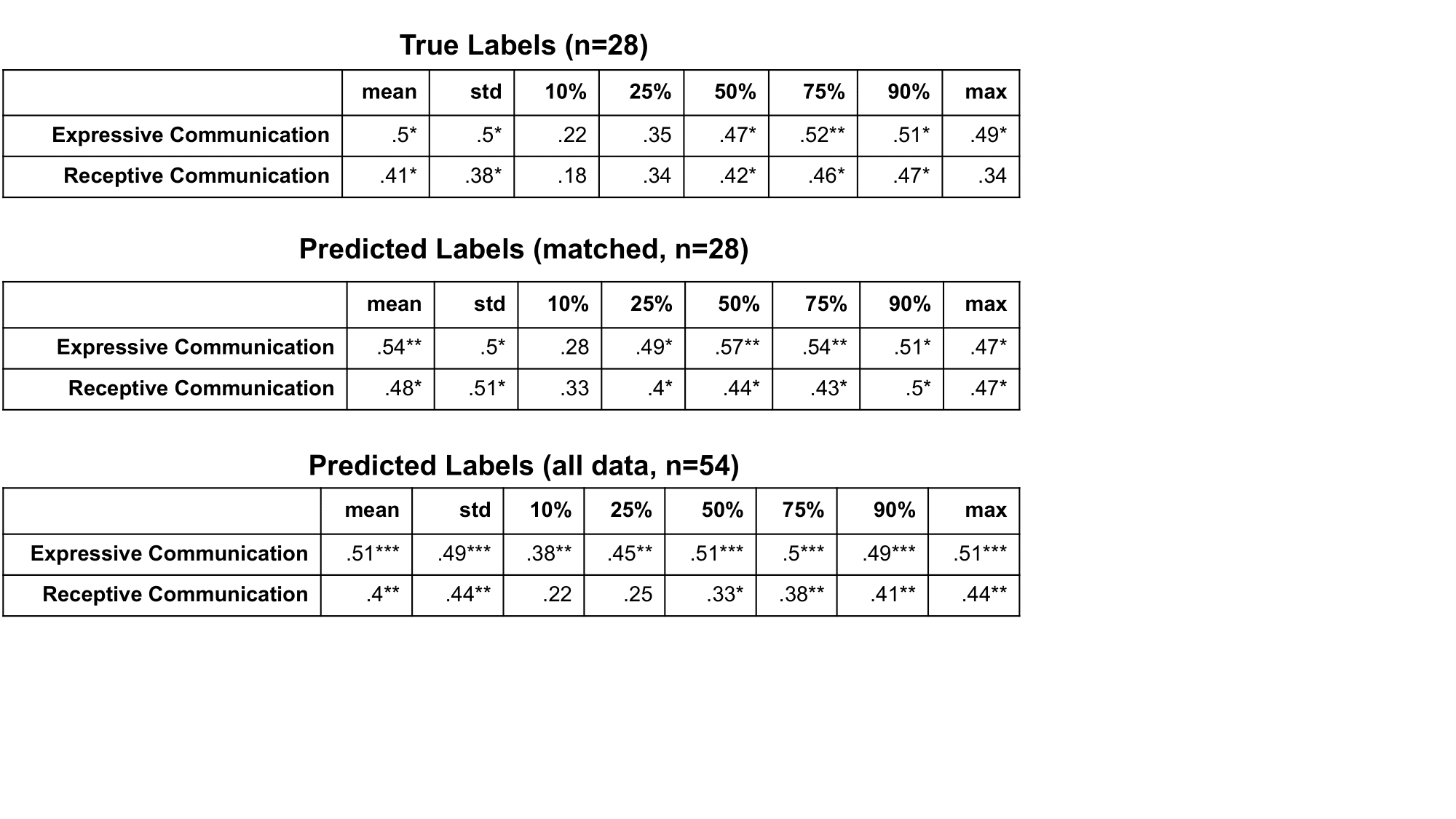
*

#### **Supplementary Table 3: Utterance Duration (UD) statistics and their correlation with the VABS communication scales.**

*The UD can be summarised using a variety of statistics. For the main paper, we showed the results using the mean UD. Here, we are showing the results from all statistics (columns) and their correlation with the VABS expressive and receptive communication scores (rows). In addition, we report the correlations for the predicted labels when using the same subset of conversations as the true labels (middle; Predicted Labels (matched, n=28)).*

*
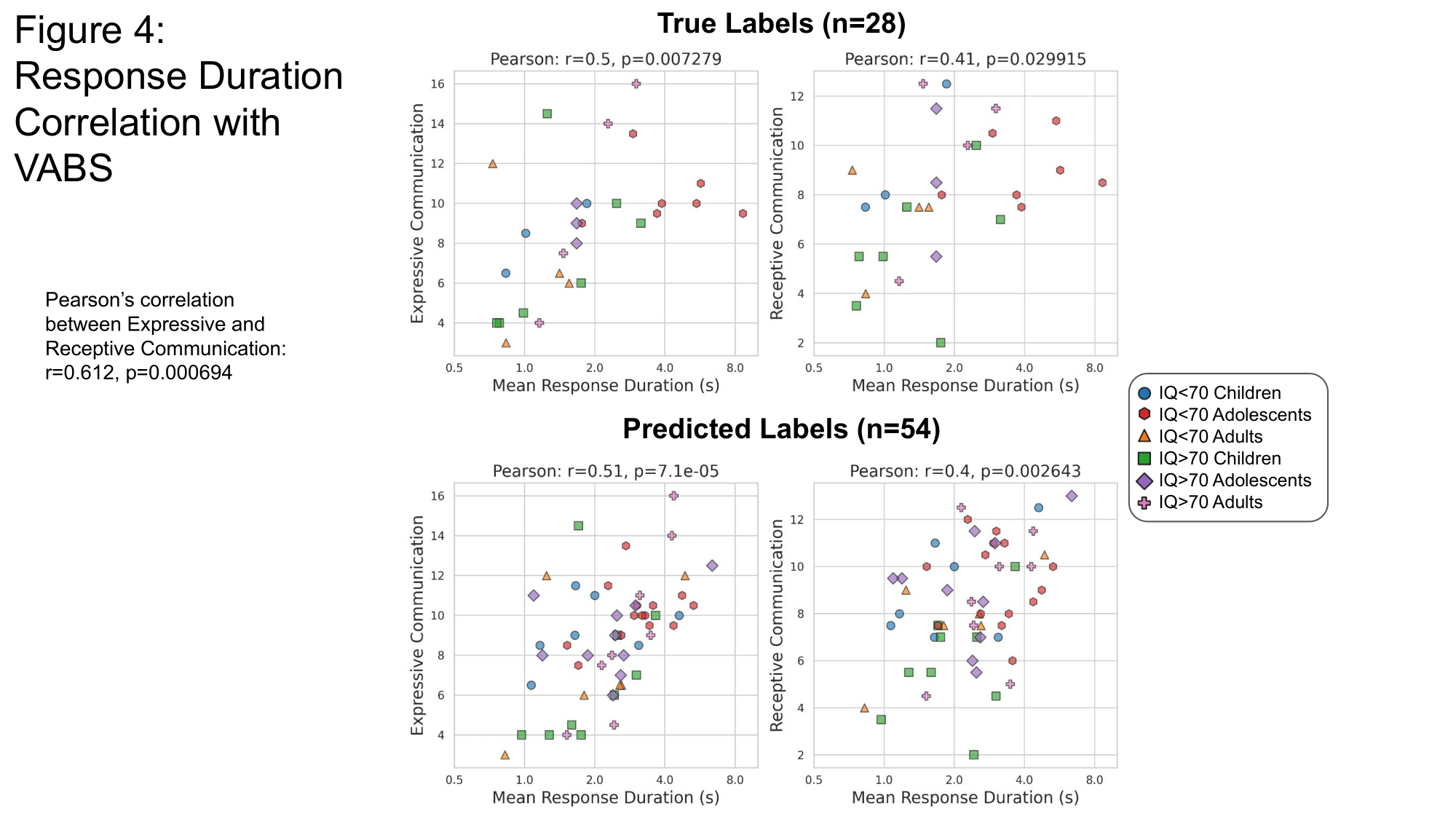
*


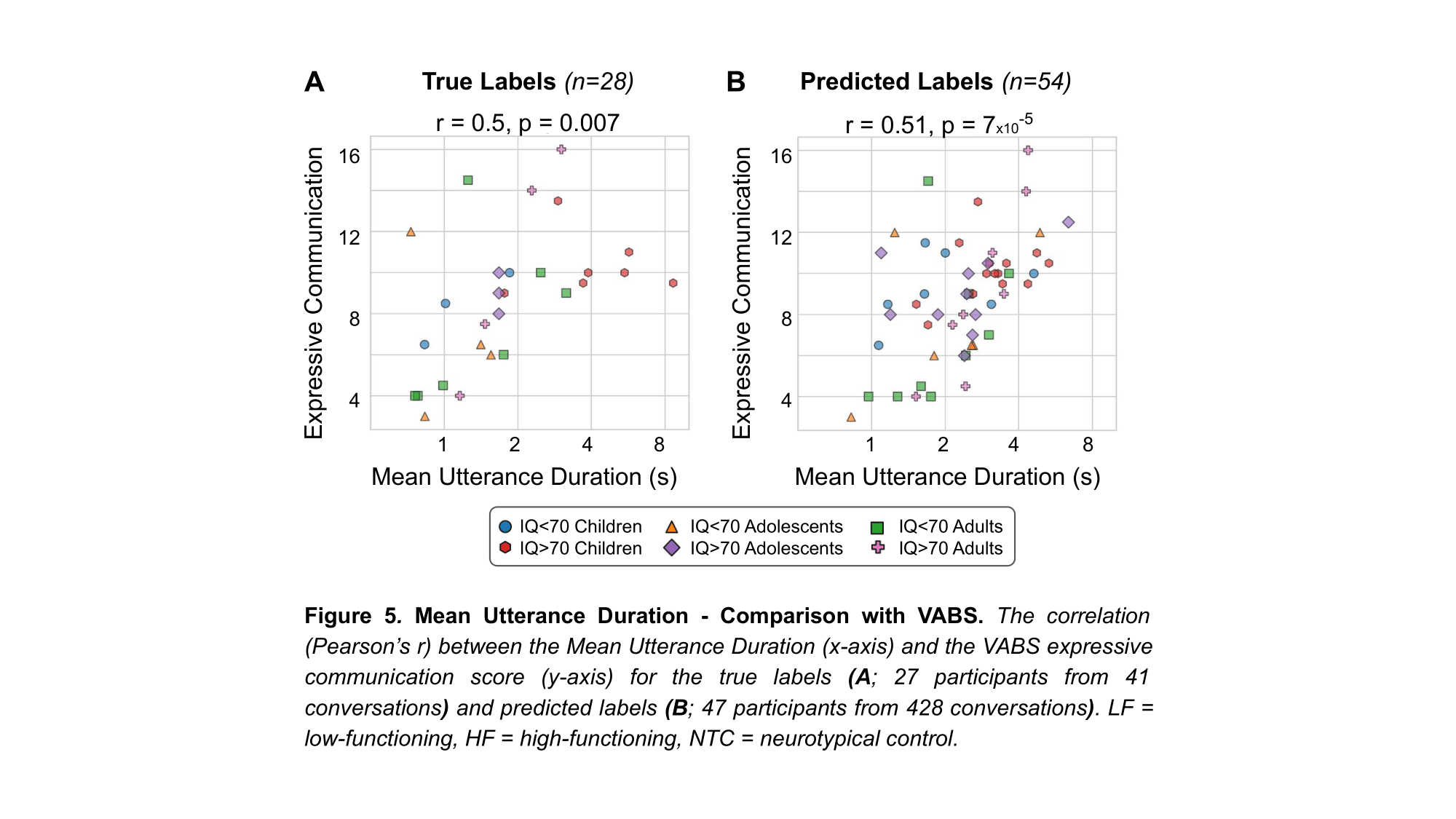


#### **Supplementary Figure 1: Mean Utterance Duration (UD) - Comparison with VABS.**

*The correlation (Pearson’s r) between the mean UD (x-axis; logarithmic scale) and the VABS expressive communication score (y-axis; left column) and the receptive communication score (right column) for the true labels (top row: 28 participants from 42 conversations****)*** *and predicted labels (bottom row: 54 participants from 499 conversations****)****. Each dot represents an ASD participant.*

*
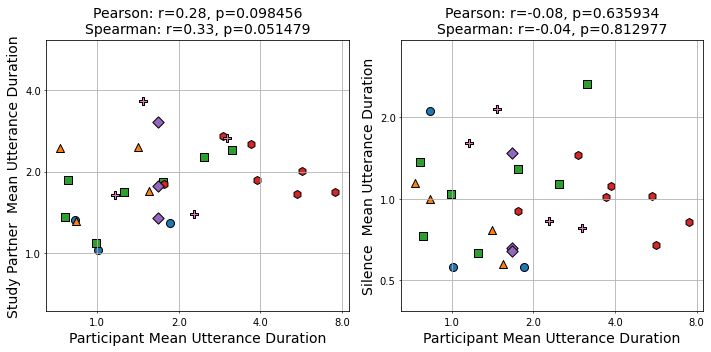
*


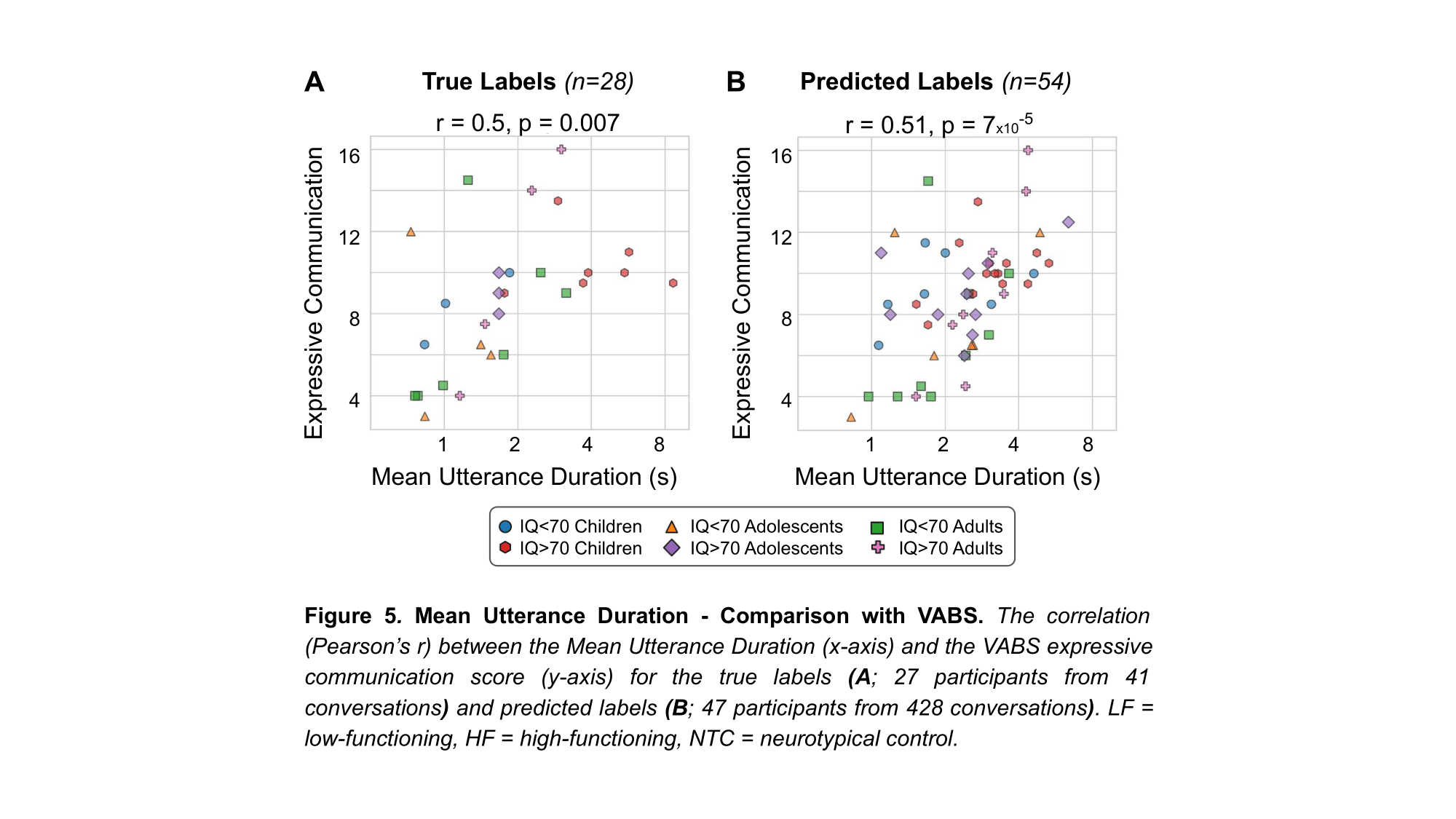


#### **Supplementary Figure 2: Mean Utterance Duration (UD) - Participant and Study Partner Comparison.**

*The correlation between the Participant’s and Study Partner’s mean UD (left) and between the Participant’s and Silence mean UD (right) using the hand-labelled data. Each dot represents an ASD participant.*

*
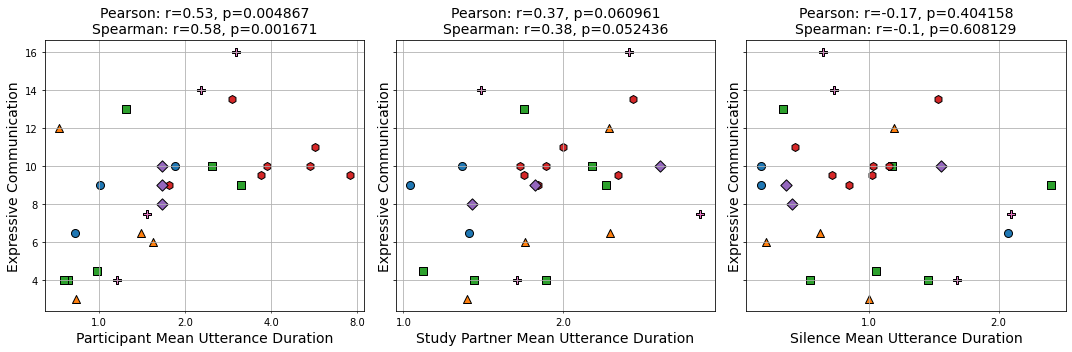
*


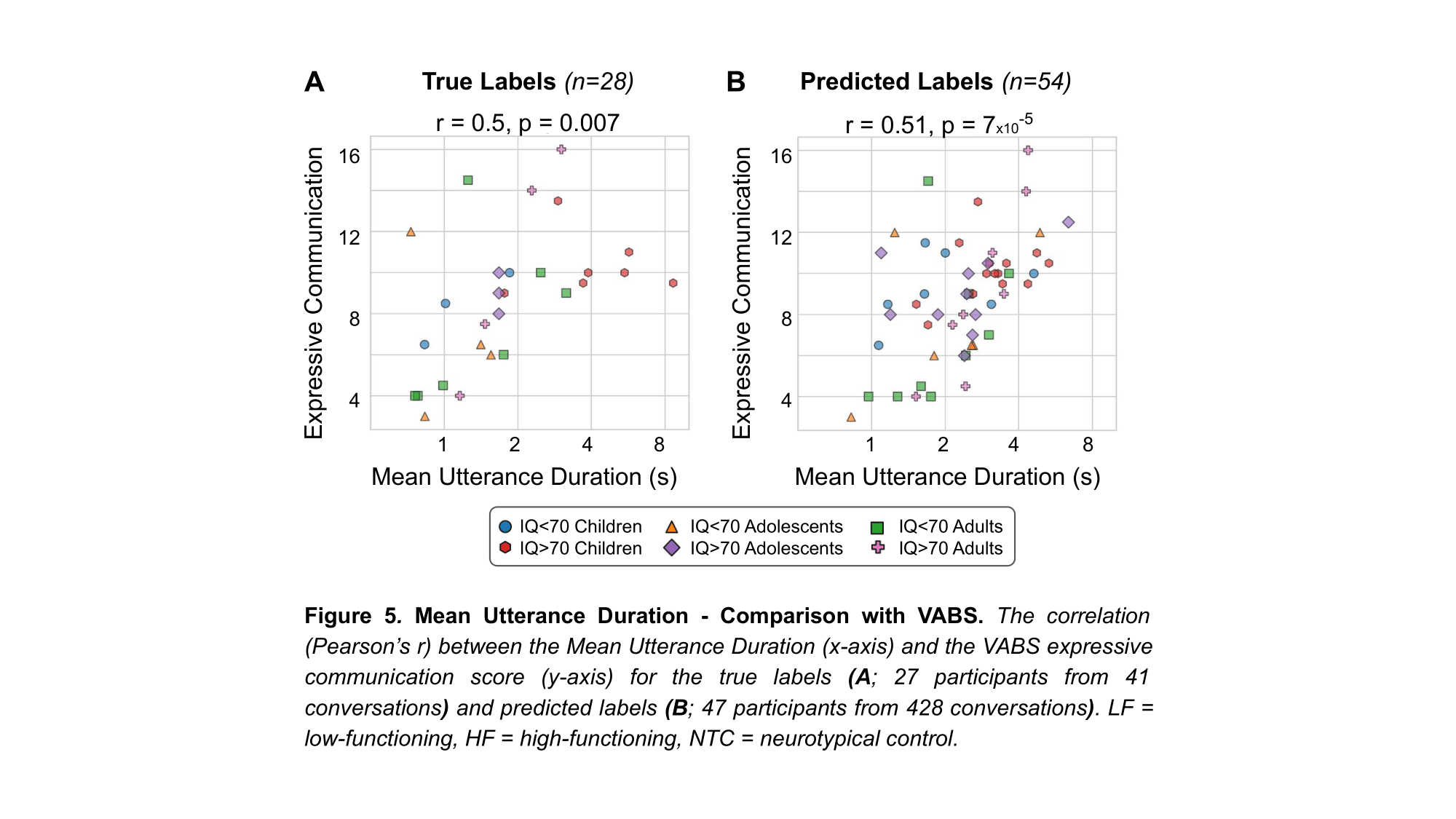


#### **Supplementary Figure 3*:* Mean Utterance Duration (UD) - Comparison with VABS.**

*The correlation (Pearson’s r) between the mean UD (x-axis; logarithmic scale) and the VABS expressive communication score (y-axis) for the participant’s speech (left), study partner speech (middle) and silence (right) using the hand-labelled data. Each dot represents an ASD participant.*


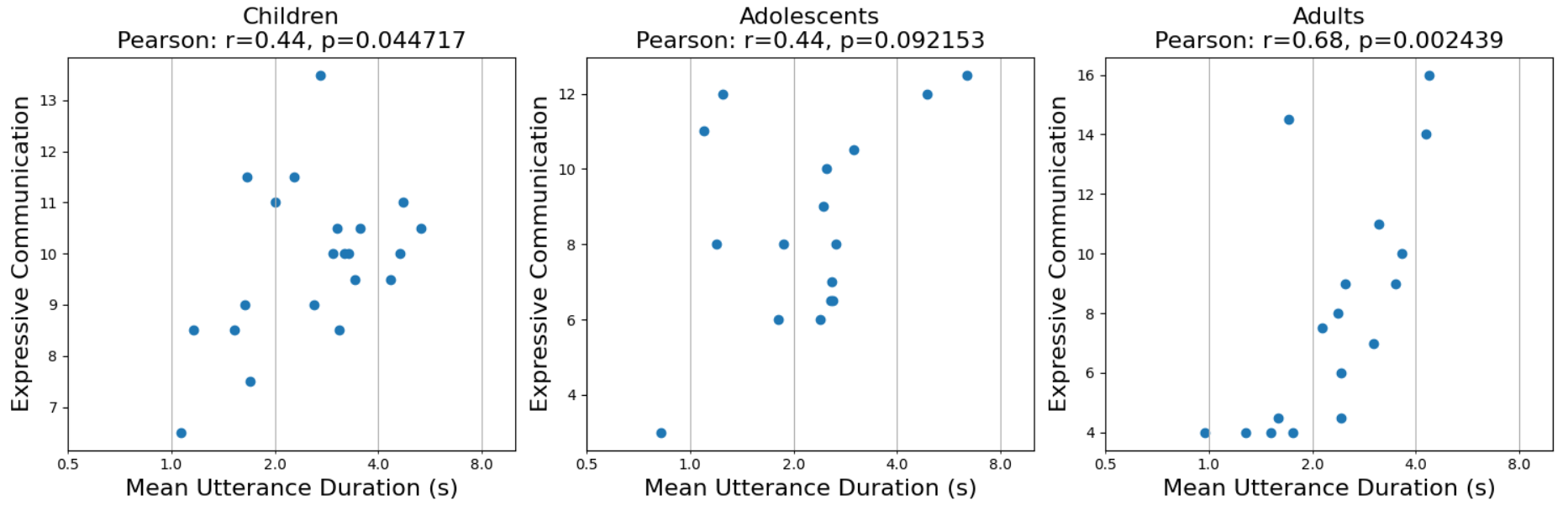


#### **Supplementary Figure 4*:* Mean Utterance Duration (UD) vs. VABS - Age Comparison.**

*The correlation (Pearson’s r) between the mean UD (x-axis; logarithmic scale) and the VABS expressive communication score (y-axis) for each age group (left: children, middle: adolescents, right: adults). Each dot represents an ASD participant.*

***Conversations > 5 minutes Conversations < 5 minutes***


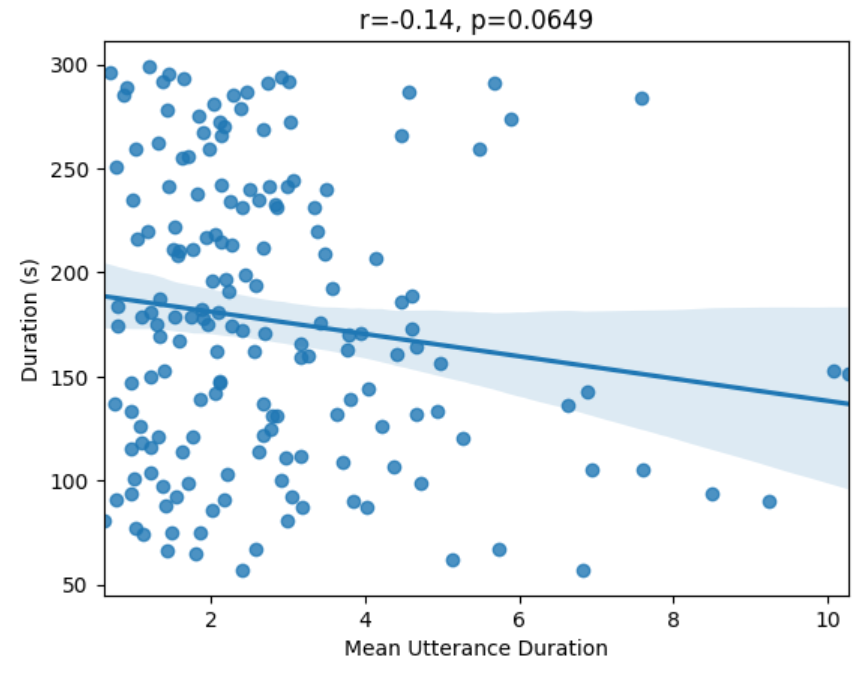


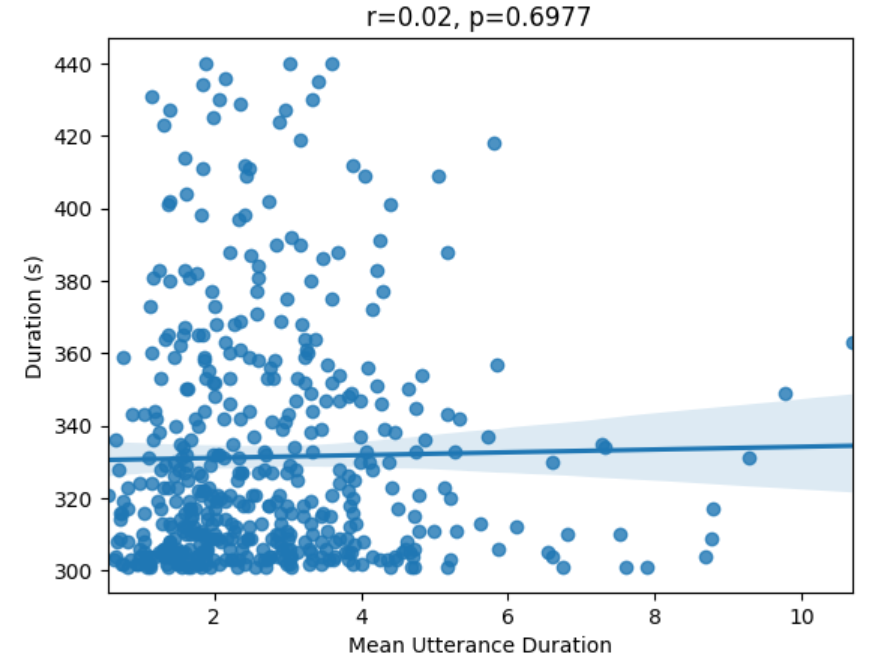


#### **Supplementary Figure 5*:* Mean Utterance Duration (UD) vs. Conversation Length.**

*The correlation (Pearson’s r) between the mean UD (x-axis) and the conversation duration (s; y-axis) for conversations that were greater than 5 minutes (left) and less than 5 minutes (right). Each dot represents a conversation.*
